# Supplementary material for: Conditional chemoconnectomics (cCCTomics) as a strategy for efficient and conditional targeting of chemical transmission
Source: eLife. 2024 Apr 30;12:RP91927. doi: 10.7554/eLife.91927 (PMC11060718; doi:10.7554/eLife.91927)
Supplement: Figure 3—source data 3. [file elife-91927-fig3-data3.zip › Figure 3 source data3 part 2.zip!/Figure 3-Source data 3-part2/1025-13-CRISPResso_Report_ack040l3.zip!/CRISPResso_on_1025_14/2b.Nucleotide_percentage_quilt_around_sgRNA_agcacaaacttggcggtgac.pdf]

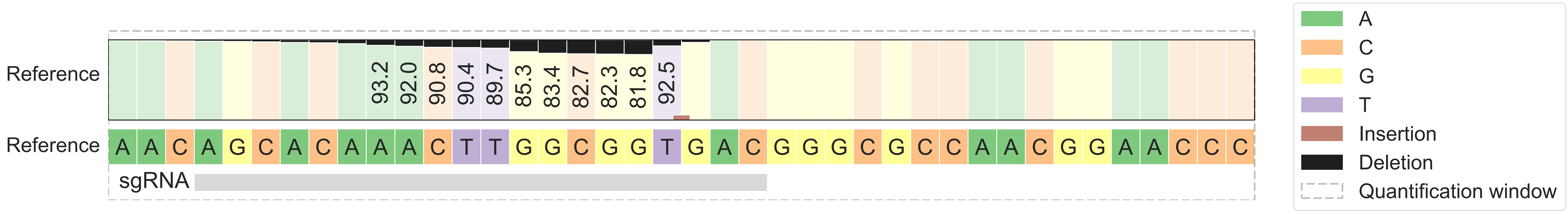
Reference
93.2
92.0
90.8
90.4
89.7
85.3
83.4
82.7
82.3
81.8
92.5
Reference
A A C A G C A C A A A C T T G G C G G T G A C G G G C G C C A A C G G A A C C C
sgRNA
A
C
G
T
Insertion
Deletion
Quantification window
